# Supplementary material for: Cu2Se Nanoparticles Encapsulated by Nitrogen-Doped Carbon Nanofibers for Efficient Sodium Storage
Source: Nanomaterials (Basel). 2020 Feb 10;10(2):302. doi: 10.3390/nano10020302 (PMC7075191; doi:10.3390/nano10020302)
Supplement: Supplementary file 1 [file nanomaterials-10-00302-s001.pdf]

Supplementary Materials: Cu_2Se Nanoparticles Encapsulated by Nitrogen-Doped Carbon Nanofibers for Efficient Sodium Storage

Le Hu [1,†], Chaoqun Shang [1,*,†], Eser Metin Akinoglu [2], Xin Wang [1,2,*] and Guofu Zhou [1,2]

1 National Center for International Research on Green Optoelectronics, South China Normal University, Guangzhou 510006, China; hule@m.scnu.edu.cn (L.H.); guofu.zhou@m.scnu.edu.cn (G.Z.)

2 International Academy of Optoelectronics at Zhaoqing, South China Normal University, Zhaoqing 526060, China; e.akinoglu@zq-scnu.org

* Correspondence: chaoqun.shang@ecs-scnu.org (C.S.); wangxin@scnu.edu.cn (X.W.)

† These authors contributed equally to this work.

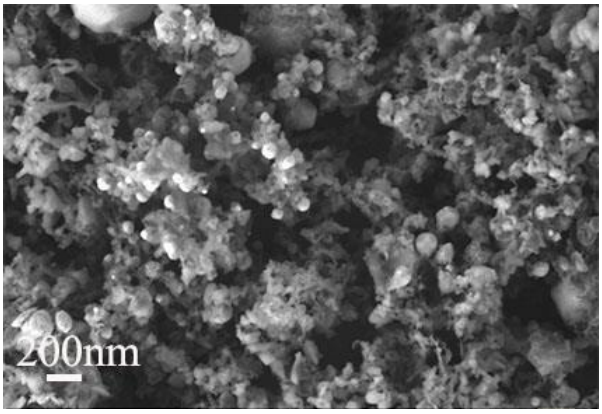

Figure S1. Typical SEM image of Cu_2Se.

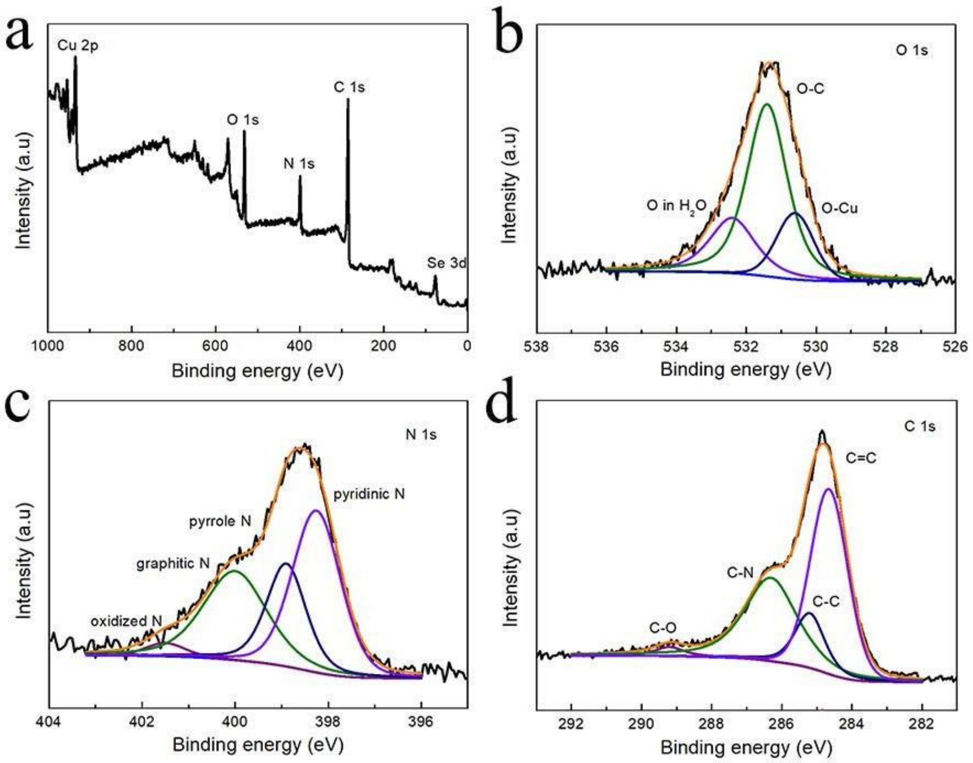

Figure S2. (**a**) High-resolution XPS survey spectra and corresponding (**b**) O 1s and (**c**) N 1s and (**d**) C 1s of the Cu_2Se-NC, respectively.

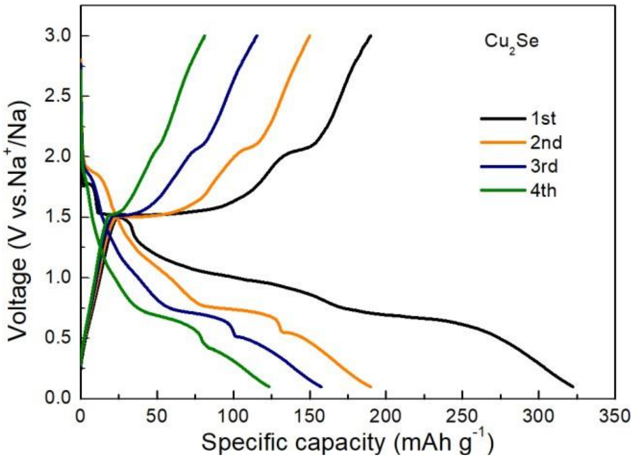

Figure S3. Charge/discharge curves of the Cu_2Se.

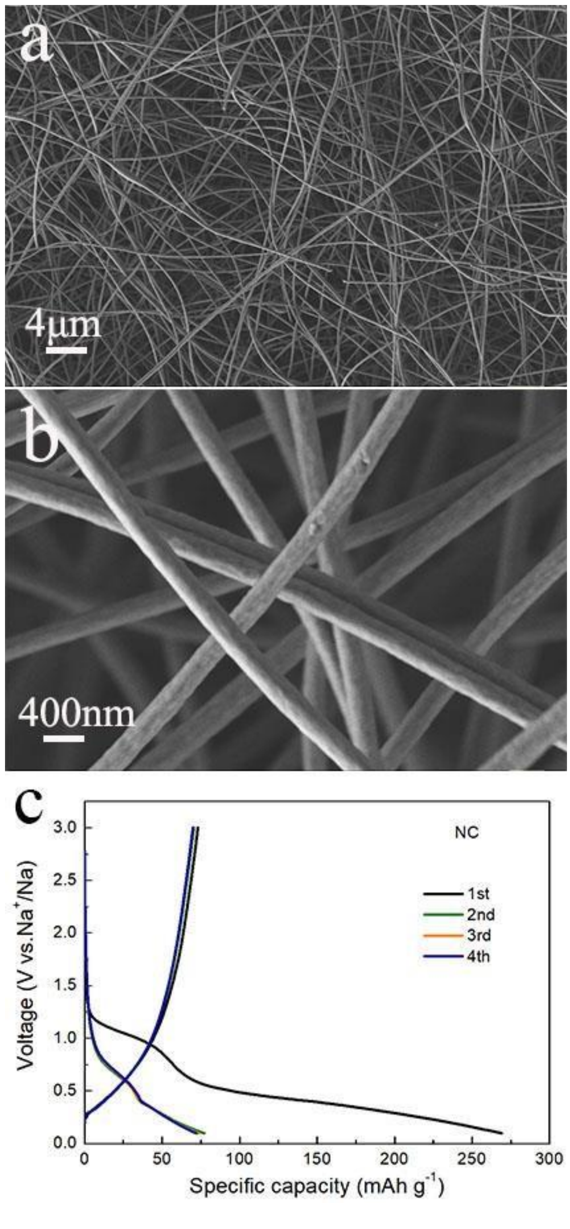

Figure S4. The SEM images and Charge/discharge curves of the NC.

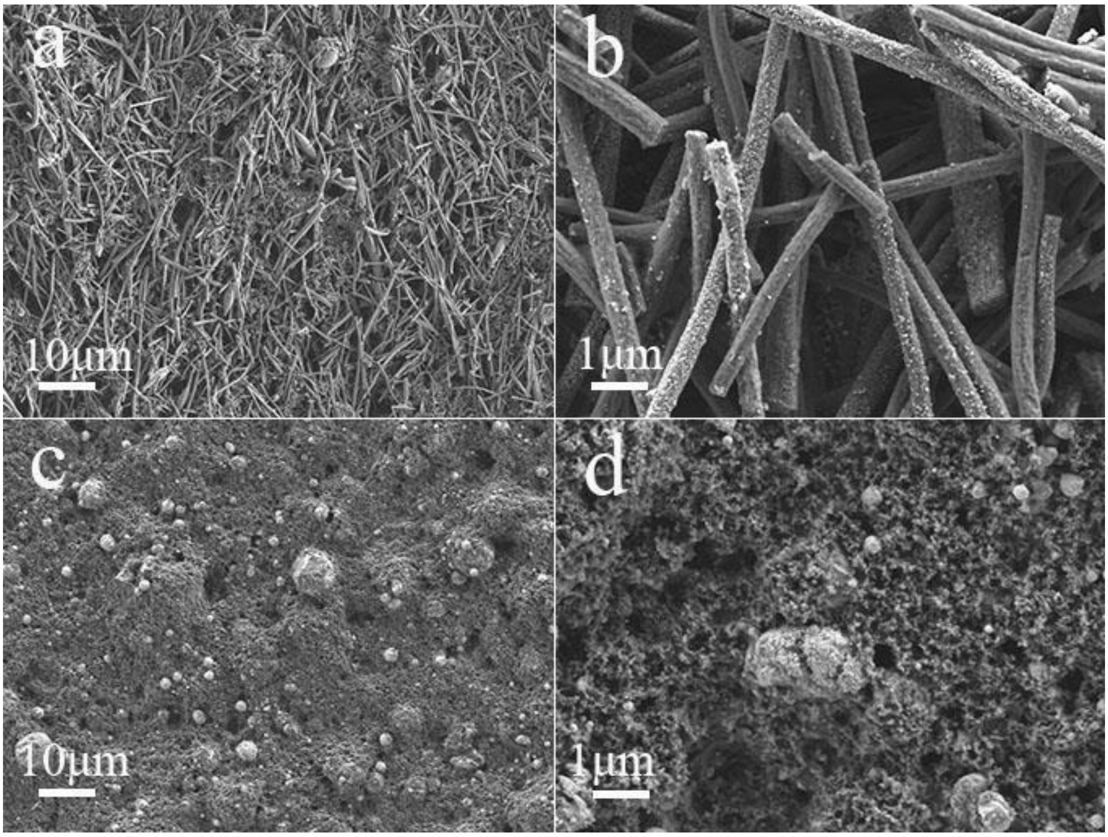

Figure S5. SEM images of (**a**, **b**) Cu_2Se-NC and (**c**, **d**) Cu_2Se electrode before cycle at a current density of 0.1 A g^{-1}.

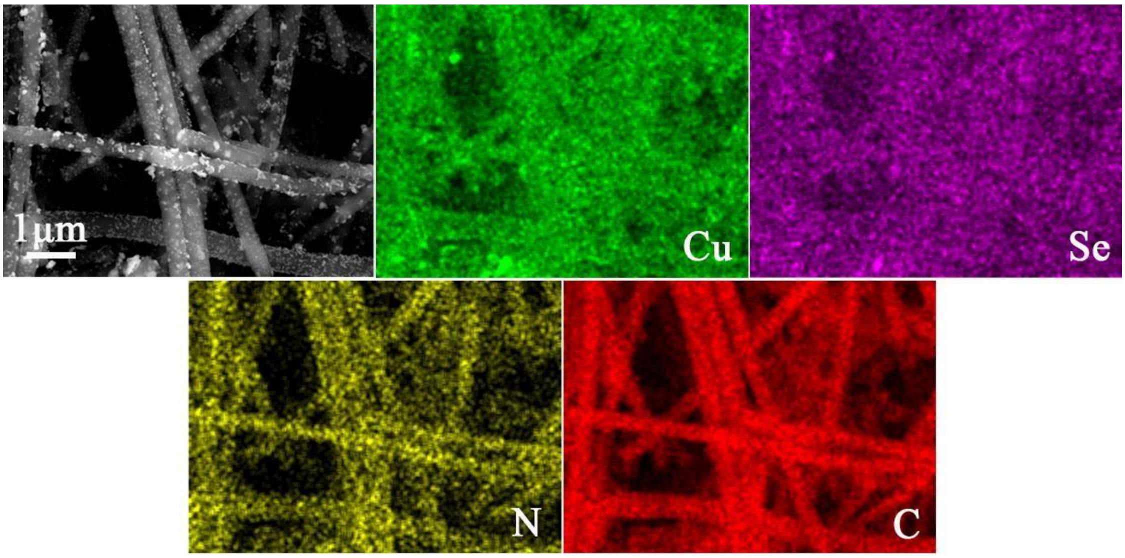

Figure S6. SEM image and corresponding elemental mappings of the cycled Cu_2Se-NC.

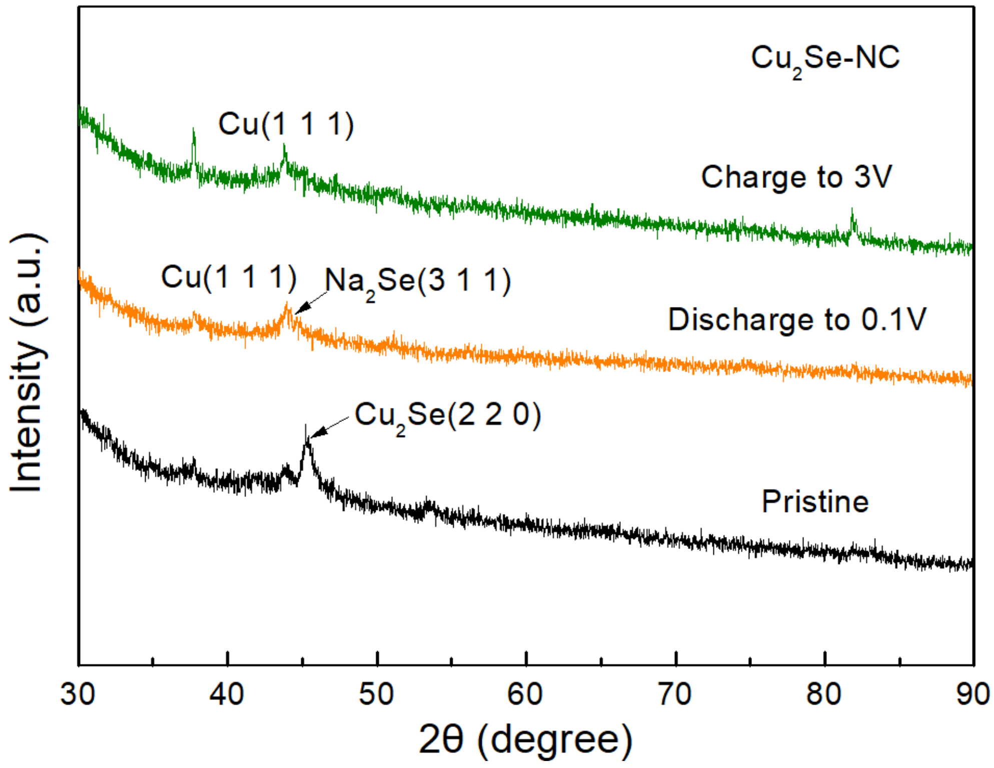

Figure S7. Ex situ XRD patterns of the Cu_2Se-NC anode under different state: pristine, discharge to 0.1 V, and charge to 3 V, respectively.
